# *catena*-Poly[[bis­[2-(1*H*-1,2,4-triazol-1-yl-κ*N* ^4^)pyrazine]cadmium(II)]-di-μ-thio­cyanato-κ^2^ *S*:*N*;κ^2^ *N*:*S*]

**DOI:** 10.1107/S1600536809027135

**Published:** 2009-07-18

**Authors:** Hong Li, Long Miao Xie

**Affiliations:** a Department of Chemistry and Chemical Engineering, Institute of Materials Chemistry, Binzhou University, Binzhou 256603, People’s Republic of China; b Department of Chemistry, Shandong Normal University, Jinan 250014, People’s Republic of China

## Abstract

The title compound, [Cd(NCS)_2_(C_6_H_5_N_5_)_2_]_*n*_, is a coordination polymer with the Cd^II^ centre located on a twofold rotation axis. The Cd^II^ centre assumes a distorted octa­hedral geometry. The thio­cyanate anions function as bridging ligands between the Cd^II^ centres, leading to a chain-like arrangement expanding along [001].

## Related literature

For a related structure, see: Yang & Shi (2008 ▶).
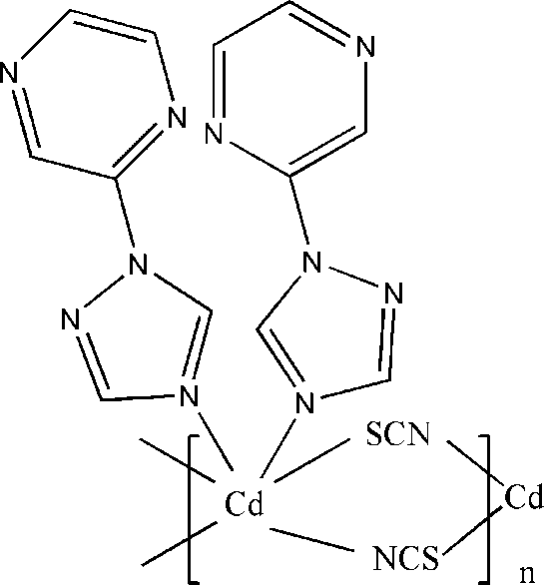


## Experimental

### 

#### Crystal data

[Cd(NCS)_2_(C_6_H_5_N_5_)_2_]
*M* *_r_* = 522.86
Monoclinic, 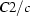

*a* = 25.818 (4) Å
*b* = 7.4077 (10) Å
*c* = 11.0276 (15) Å
β = 113.843 (2)°
*V* = 1929.1 (5) Å^3^
*Z* = 4
Mo *K*α radiation
μ = 1.38 mm^−1^
*T* = 298 K
0.41 × 0.21 × 0.20 mm

#### Data collection

Bruker SMART APEX CCD diffractometer
Absorption correction: multi-scan (*SADABS*; Bruker, 1997 ▶) *T*_min_ = 0.602, *T*_max_ = 0.770
5381 measured reflections
2085 independent reflections
2005 reflections with *I* > 2σ(*I*)
*R*_int_ = 0.022

#### Refinement

*R*[*F* ^2^ > 2σ(*F* ^2^)] = 0.022
*wR*(*F* ^2^) = 0.058
*S* = 1.10
2085 reflections
133 parameters
H-atom parameters constrained
Δρ_max_ = 0.35 e Å^−3^
Δρ_min_ = −0.47 e Å^−3^

### 

Data collection: *SMART* (Bruker, 1997 ▶); cell refinement: *SAINT* (Bruker, 1997 ▶); data reduction: *SAINT*; program(s) used to solve structure: *SHELXS97* (Sheldrick, 2008 ▶); program(s) used to refine structure: *SHELXL97* (Sheldrick, 2008 ▶); molecular graphics: *SHELXTL* (Sheldrick, 2008 ▶); software used to prepare material for publication: *SHELXTL*.

## Supplementary Material

Crystal structure: contains datablocks I, global. DOI: 10.1107/S1600536809027135/bt2992sup1.cif

Structure factors: contains datablocks I. DOI: 10.1107/S1600536809027135/bt2992Isup2.hkl

Additional supplementary materials:  crystallographic information; 3D view; checkCIF report

## Figures and Tables

**Fig. 1.**
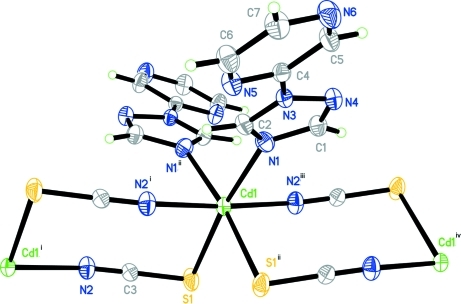
Coordination around the Cd atom with the atom-numbering scheme. Displacement ellipsoids are drawn at the 30% probability level. [Symmetry codes: (i) -x + 1, -y, -z + 1 (ii) -x + 1, y, -z + 3/2 (iii) x, -y, z + 1/2 (iv) -x + 1, -y, -z + 2]

**Fig. 2.**
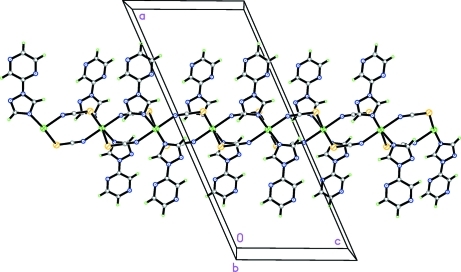
Packing diagram of the title compound.

## supplementary crystallographic information

### Comment

2-(1*H*-1,2,4-triazol-1-yl)pyrazine is similar to 2-(pyrazol-1-yl)pyrazine (Yang & Shi, 2008) and therefore it should act as a brdiging ligand. We are interested in synthesizing complexes with mixed bridging ligands and selected thiocyanato and 2-(1*H*-1,2,4-triazol-1-yl)pyrazine as ligands. However, 2-(1*H*-1,2,4-triazol-1-yl)pyrazine only functions as a terminal ligand.

The coordination geometry of the Cd centres is shown in Fig. 1. The Cd atom is in a distorted octahedral CdN_4_S_2_ coordination geometry. In the crystal each Cd^II^ ion is surrounded by two other symmetry-related Cd^II^ ions with separation with 5.7105 (7) Å and the adjacent Cd^II^ ions were bridged by two thiocyanato anions and it forms a one-dimensional chain along the *c* axis. 2-(1*H*-1,2,4-triazol-1-yl)pyrazine only acts as a monodentate ligand.

### Experimental

6 ml methanol solution of 2-(1*H*-1,2,4-triazol-1-yl)pyrazine (0.0345 g, 0.191 mmol), 5 ml C d(ClO_4_)_2_.6H_2_O (0.0809 g, 0.193 mmol) H_2_O solution and 5 ml NaSCN (0.0315 g, 0.389 mmol) H_2_O solution were mixed together and stirred for a few minutes. The colorless single crystals were obtained after the filtrate had been allowed to stand at room temperature for two weeks.

### Refinement

All H atoms were placed in calculated positions and refined as riding with C—H = 0.93 Å and *U*_iso_(H) = 1.2*U*_eq_(C).

### Crystal data

**Table:**

[Cd(NCS)_2_(C_6_H_5_N_5_)_2_]	*F*(000) = 1032
*M**_r_* = 522.86	*D*_x_ = 1.800 Mg m^−^^3^
Monoclinic, *C*2/*c*	Mo *K*α radiation, λ = 0.71073 Å
Hall symbol: -C 2yc	Cell parameters from 4306 reflections
*a* = 25.818 (4) Å	θ = 2.9–28.3°
*b* = 7.4077 (10) Å	µ = 1.38 mm^−^^1^
*c* = 11.0276 (15) Å	*T* = 298 K
β = 113.843 (2)°	Block, colourless
*V* = 1929.1 (5) Å^3^	0.41 × 0.21 × 0.20 mm
*Z* = 4	

### Data collection

**Table:**

Bruker SMART APEX CCD diffractometer	2085 independent reflections
Radiation source: fine-focus sealed tube	2005 reflections with *I* > 2σ(*I*)
graphite	*R*_int_ = 0.022
φ and ω scans	θ_max_ = 27.0°, θ_min_ = 1.7°
Absorption correction: multi-scan (*SADABS*; Bruker, 1997)	*h* = −32→23
*T*_min_ = 0.602, *T*_max_ = 0.770	*k* = −7→9
5381 measured reflections	*l* = −10→13

### Refinement

**Table:**

Refinement on *F*^2^	Secondary atom site location: difference Fourier map
Least-squares matrix: full	Hydrogen site location: inferred from neighbouring sites
*R*[*F*^2^ > 2σ(*F*^2^)] = 0.022	H-atom parameters constrained
*wR*(*F*^2^) = 0.058	*w* = 1/[σ^2^(*F*_o_^2^) + (0.0298*P*)^2^ + 1.3229*P*] where *P* = (*F*_o_^2^ + 2*F*_c_^2^)/3
*S* = 1.10	(Δ/σ)_max_ = 0.002
2085 reflections	Δρ_max_ = 0.35 e Å^−^^3^
133 parameters	Δρ_min_ = −0.47 e Å^−^^3^
0 restraints	Extinction correction: *SHELXL97* (Sheldrick, 2008), Fc^*^=kFc[1+0.001xFc^2^λ^3^/sin(2θ)]^-1/4^
Primary atom site location: structure-invariant direct methods	Extinction coefficient: 0.0082 (3)

### Special details

**Table:**

Geometry. All e.s.d.'s (except the e.s.d. in the dihedral angle between two l.s. planes) are estimated using the full covariance matrix. The cell e.s.d.'s are taken into account individually in the estimation of e.s.d.'s in distances, angles and torsion angles; correlations between e.s.d.'s in cell parameters are only used when they are defined by crystal symmetry. An approximate (isotropic) treatment of cell e.s.d.'s is used for estimating e.s.d.'s involving l.s. planes.
Refinement. Refinement of *F*^2^ against ALL reflections. The weighted *R*-factor *wR* and goodness of fit *S* are based on *F*^2^, conventional *R*-factors *R* are based on *F*, with *F* set to zero for negative *F*^2^. The threshold expression of *F*^2^ > σ(*F*^2^) is used only for calculating *R*-factors(gt) *etc*. and is not relevant to the choice of reflections for refinement. *R*-factors based on *F*^2^ are statistically about twice as large as those based on *F*, and *R*- factors based on ALL data will be even larger.

### Fractional atomic coordinates and isotropic or equivalent isotropic displacement parameters (Å^2^)

**Table:**

	*x*	*y*	*z*	*U*_iso_*/*U*_eq_	
C1	0.43493 (9)	0.3626 (3)	0.9002 (2)	0.0392 (5)	
H1	0.4636	0.3440	0.9838	0.047*	
C2	0.39121 (9)	0.3614 (3)	0.6932 (2)	0.0382 (4)	
H2	0.3808	0.3457	0.6027	0.046*	
C3	0.56274 (8)	−0.1295 (3)	0.5829 (2)	0.0334 (4)	
C4	0.30256 (8)	0.5013 (3)	0.68395 (18)	0.0322 (4)	
C5	0.27688 (11)	0.5921 (3)	0.7544 (2)	0.0455 (5)	
H5	0.2974	0.6177	0.8437	0.055*	
C6	0.22301 (10)	0.5174 (4)	0.4984 (2)	0.0493 (5)	
H6	0.2029	0.4949	0.4084	0.059*	
C7	0.19638 (11)	0.6044 (3)	0.5671 (3)	0.0505 (6)	
H7	0.1586	0.6371	0.5226	0.061*	
Cd1	0.5000	0.10030 (3)	0.7500	0.03107 (10)	
N1	0.43983 (7)	0.3119 (2)	0.78646 (17)	0.0391 (4)	
N2	0.55550 (9)	−0.1113 (3)	0.47410 (19)	0.0455 (5)	
N3	0.35900 (7)	0.4377 (2)	0.74783 (16)	0.0317 (3)	
N4	0.38687 (8)	0.4394 (2)	0.88241 (17)	0.0377 (4)	
N5	0.27701 (8)	0.4642 (3)	0.55708 (17)	0.0422 (4)	
N6	0.22297 (9)	0.6433 (3)	0.6954 (2)	0.0535 (5)	
S1	0.57240 (3)	−0.15916 (10)	0.73782 (5)	0.05367 (18)	

### Atomic displacement parameters (Å^2^)

**Table:**

	*U*^11^	*U*^22^	*U*^33^	*U*^12^	*U*^13^	*U*^23^
C1	0.0367 (11)	0.0455 (11)	0.0340 (10)	−0.0001 (9)	0.0129 (9)	−0.0018 (9)
C2	0.0371 (11)	0.0444 (11)	0.0356 (10)	0.0028 (9)	0.0172 (9)	−0.0052 (8)
C3	0.0287 (9)	0.0379 (10)	0.0307 (10)	0.0051 (7)	0.0089 (8)	−0.0017 (7)
C4	0.0329 (9)	0.0331 (9)	0.0335 (10)	0.0004 (7)	0.0162 (8)	0.0008 (7)
C5	0.0460 (13)	0.0563 (14)	0.0358 (11)	0.0117 (10)	0.0181 (10)	−0.0034 (9)
C6	0.0409 (12)	0.0649 (15)	0.0373 (11)	0.0040 (11)	0.0106 (9)	−0.0060 (10)
C7	0.0370 (12)	0.0666 (16)	0.0452 (14)	0.0126 (10)	0.0138 (11)	0.0007 (10)
Cd1	0.03018 (13)	0.03752 (14)	0.02687 (13)	0.000	0.01294 (9)	0.000
N1	0.0349 (9)	0.0439 (10)	0.0404 (9)	0.0014 (7)	0.0171 (7)	−0.0046 (8)
N2	0.0428 (10)	0.0607 (12)	0.0319 (10)	0.0058 (8)	0.0141 (8)	0.0017 (8)
N3	0.0321 (8)	0.0356 (8)	0.0300 (8)	−0.0009 (6)	0.0153 (7)	−0.0026 (6)
N4	0.0367 (9)	0.0465 (9)	0.0302 (9)	0.0006 (7)	0.0138 (7)	−0.0024 (7)
N5	0.0369 (9)	0.0549 (10)	0.0349 (9)	0.0035 (8)	0.0146 (7)	−0.0061 (8)
N6	0.0476 (11)	0.0696 (13)	0.0450 (11)	0.0190 (10)	0.0204 (9)	−0.0017 (10)
S1	0.0699 (4)	0.0614 (4)	0.0296 (3)	0.0323 (3)	0.0201 (3)	0.0093 (2)

### Geometric parameters (Å, °)

**Table:**

C1—N4	1.306 (3)	C6—N5	1.337 (3)
C1—N1	1.363 (3)	C6—C7	1.372 (3)
C1—H1	0.9300	C6—H6	0.9300
C2—N1	1.313 (3)	C7—N6	1.331 (3)
C2—N3	1.334 (3)	C7—H7	0.9300
C2—H2	0.9300	Cd1—N2^i^	2.3031 (19)
C3—N2	1.145 (3)	Cd1—N2^ii^	2.3031 (19)
C3—S1	1.639 (2)	Cd1—N1^iii^	2.3528 (18)
C4—N5	1.312 (3)	Cd1—N1	2.3528 (17)
C4—C5	1.383 (3)	Cd1—S1^iii^	2.7220 (6)
C4—N3	1.418 (2)	Cd1—S1	2.7220 (6)
C5—N6	1.331 (3)	N2—Cd1^ii^	2.3031 (19)
C5—H5	0.9300	N3—N4	1.363 (2)
			
N4—C1—N1	114.71 (19)	N2^ii^—Cd1—N1	89.56 (7)
N4—C1—H1	122.6	N1^iii^—Cd1—N1	96.47 (9)
N1—C1—H1	122.6	N2^i^—Cd1—S1^iii^	96.54 (5)
N1—C2—N3	109.75 (19)	N2^ii^—Cd1—S1^iii^	86.34 (5)
N1—C2—H2	125.1	N1^iii^—Cd1—S1^iii^	173.10 (5)
N3—C2—H2	125.1	N1—Cd1—S1^iii^	87.01 (5)
N2—C3—S1	178.9 (2)	N2^i^—Cd1—S1	86.34 (5)
N5—C4—C5	123.5 (2)	N2^ii^—Cd1—S1	96.54 (5)
N5—C4—N3	115.64 (17)	N1^iii^—Cd1—S1	87.01 (5)
C5—C4—N3	120.82 (18)	N1—Cd1—S1	173.10 (5)
N6—C5—C4	120.5 (2)	S1^iii^—Cd1—S1	90.16 (4)
N6—C5—H5	119.7	C2—N1—C1	103.22 (18)
C4—C5—H5	119.7	C2—N1—Cd1	122.72 (14)
N5—C6—C7	121.9 (2)	C1—N1—Cd1	130.85 (14)
N5—C6—H6	119.1	C3—N2—Cd1^ii^	153.59 (19)
C7—C6—H6	119.1	C2—N3—N4	110.16 (17)
N6—C7—C6	122.0 (2)	C2—N3—C4	128.28 (17)
N6—C7—H7	119.0	N4—N3—C4	121.46 (16)
C6—C7—H7	119.0	C1—N4—N3	102.16 (16)
N2^i^—Cd1—N2^ii^	175.94 (10)	C4—N5—C6	115.50 (19)
N2^i^—Cd1—N1^iii^	89.56 (7)	C7—N6—C5	116.5 (2)
N2^ii^—Cd1—N1^iii^	87.73 (7)	C3—S1—Cd1	97.98 (7)
N2^i^—Cd1—N1	87.73 (7)		

Symmetry codes: (i) *x*, −*y*, *z*+1/2; (ii) −*x*+1, −*y*, −*z*+1; (iii) −*x*+1, *y*, −*z*+3/2.
